# Triboelectric Nanogenerator-Based Stretch Sensor

**DOI:** 10.3390/s26206493

**Published:** 2026-10-14

**Authors:** Yafei Wang, Lei Shi, Miaomiao Zhong, Lihua Tang, Wee Chen Gan, Yuting Zhu, Kean Aw

**Affiliations:** 1 Department of Mechanical and Mechatronics Engineering, University of Auckland, Auckland 1010, New Zealand; nway947@aucklanduni.ac.nz (Y.W.); lshi378@aucklanduni.ac.nz (L.S.); l.tang@auckland.ac.nz (L.T.); 2 Shien-Ming Wu School of Intelligent Engineering, South China University of Technology, Guangzhou 510641, China; 202510192828@mail.scut.edu.cn; 3 New Energy Science and Engineering, Xiamen University Malaysia, Sepang 43900, Selangor, Malaysia; wcgan@xmu.edu.my; 4 School of Engineering and Built Environment, Griffith University, Brisbane, QLD 4111, Australia; y.zhu2@griffith.edu.au

**Keywords:** TENG, stretch sensor, multiple-triangular shape structure, elastomer

## Abstract

Triboelectric nanogenerators (TENGs) have been extensively investigated as both energy harvesters and self-powered sensors. While their application as force sensors has been widely reported, the use of TENGs as stretch sensors remains relatively unexplored. Stretch sensors are important for monitoring human joint and limb movements, with significant potential in rehabilitation applications such as post-stroke therapy and recovery following knee surgery. In this work, we present a preliminary TENG-based stretch sensor comprising a multi-triangular elastomer structure and an arched copper electrode. The primary objective is to establish the viability of the sensing concept and validate its fundamental operating principle. The device generates an output voltage that is proportional to the bending angle. This behavior arises from the increased contact and friction between the elastomer (Dragon Skin silicone) and copper layers as deformation increases, resulting in enhanced triboelectric charge generation and a higher output voltage. Owing to the inherent capacitive characteristics of the TENG under high-impedance loading conditions, the generated voltage can be retained for several seconds after the bending motion ceases. This retention time is sufficient for signal acquisition and joint-motion detection electronics. Experimental results demonstrate that the proposed TENG-based stretch sensor can reliably measure bending angles under both quasi-static and dynamic operating conditions, highlighting its potential for wearable motion-monitoring and rehabilitation systems.

## 1. Introduction

Stretch sensors play an important role in quantifying the movement of human limbs and joints. Such measurements are particularly valuable in rehabilitation, where continuous monitoring of joint motion can support the recovery of stroke patients and individuals undergoing rehabilitation after knee surgery. Owing to their flexibility, conformability, and ability to provide real-time measurements, stretch sensors have become attractive components in wearable systems for monitoring a wide range of human activities. These include large-scale body movements, such as those of the elbow, knee, and ankle, as well as subtle motions involving the mouth and eyes [1,2,3,4,5]. Among the available technologies, resistive and capacitive sensors are currently the most widely used for wearable motion monitoring and tactile sensing. Resistive sensors operate by changing their electrical resistance under mechanical deformation, while capacitive sensors rely on variations in capacitance induced by stretching. In general, piezoresistive sensors offer a wide sensing range and high strain sensitivity, whereas capacitive sensors provide excellent sensitivity for detecting small pressures and subtle deformations [6,7,8,9]. However, both approaches have inherent limitations. Piezoresistive sensors often suffer from hysteresis and limited repeatability, while capacitive sensors can be affected by parasitic capacitance and environmental interference [10,11]. Furthermore, both are passive sensing elements that require additional electronics to convert changes in resistance or capacitance into measurable voltage signals. This increases system complexity, hinders device miniaturization, and may compromise long-term wearability and user comfort [2,12].

In parallel with the rapid expansion of wearable technologies, substantial progress has been made in electrically functional materials for energy storage, flexible electronics, and intelligent sensing systems. Recent research has shown that device performance is increasingly governed by the interplay between environmental adaptability, interfacial regulation, and electrical response. For wearable applications, sensors are frequently exposed to complex operating conditions, including mechanical deformation, temperature fluctuations, humidity changes, and repeated loading cycles. Consequently, the development of functional materials that can maintain stable electrical characteristics under varying environmental conditions has become a major research focus. Interfacial engineering strategies, such as surface functionalization, heterostructure design, defect regulation, and charge-transport optimization, have been widely adopted to enhance electrical conductivity, charge-transfer efficiency, sensitivity, and long-term operational stability. These advances have enabled the development of flexible energy-storage devices, wearable electronic systems, and high-performance sensors with improved durability, adaptability, and reliability under real-world conditions.

The rapid growth of wearable electronics for applications in the Internet of Things (IoT), robotics, and healthcare monitoring has driven interest in alternative sensing technologies. Among these, triboelectric nanogenerators (TENGs) have attracted considerable attention for their lightweight, low-cost, high-sensitivity, and self-powered operation [1,2,12,13]. Unlike conventional resistive and capacitive sensors, TENGs function as active sensors that directly generate electrical signals in response to mechanical stimuli, eliminating the need for signal-conversion mechanisms. In addition, their self-powered nature offers the potential to reduce or even eliminate dependence on external power supplies, an important advantage for future wearable systems. TENGs operate through the combined effects of triboelectrification and electrostatic induction, in which repeated contact and separation between two materials generate surface charges that produce an electrical output [2,9,14]. Consequently, TENG-based sensors provide a promising pathway toward simpler, more compact, and potentially self-sustaining sensing systems.

In wearable sensing applications, interfacial regulation plays a particularly important role because electrical signals are often generated through interactions occurring at material interfaces. The ability to control charge generation, transfer, storage, and dissipation directly influences sensor sensitivity, signal stability, response speed, and environmental robustness. Recent studies have demonstrated that carefully engineered interfaces can significantly improve electrical responsiveness while reducing the effects of environmental disturbances, thereby enabling accurate signal acquisition during dynamic and quasi-static human motions [15,16,17,18,19]. These developments highlight a broader trend toward multifunctional material systems that combine mechanical flexibility, environmental tolerance, and stable electrical outputs, providing valuable design principles for next-generation self-powered wearable sensors. Accordingly, the concepts of interfacial charge regulation and environmental adaptability are highly relevant to triboelectric sensing technologies, where device performance is intrinsically linked to charge generation and retention at contacting material surfaces.

To date, most TENG-based sensors have been developed for force-related measurements, including tactile sensing, pressure detection and vibration monitoring [1,5,12]. These sensors are particularly attractive because of their high sensitivity and rapid response under dynamic loading conditions. For example, Chen et al. developed a dual-mode flexible pressure sensor with a triboelectric-mode sensitivity of 0.25 kPa^−1^ [20]. Similarly, Xia et al. introduced a charge-excitation strategy that improved output voltage by more than 25 times under quasi-static conditions and 15 times under dynamic operation [21]. Other flexible triboelectric sensors have demonstrated impressive performance in applications such as posture recognition, pulse monitoring, and joint-motion tracking, with response times as short as 8 ms, peak sensitivities of 174.69 V/kPa, and stable operation over more than 30,000 cycles [22].

Despite these advances, the application of TENGs as stretch sensors remains relatively unexplored. One reason is the widespread perception that triboelectric sensors can only produce meaningful electrical outputs under dynamic conditions, such as repeated contact, separation, or sliding. In this work, we propose a TENG-based stretch sensor that combines a multi-triangular elastomer structure with an arched copper electrode. As the elastomer layer, integrated with carbon-black electrodes, is stretched during bending, the triangular structures slide beneath the copper arch, generating triboelectric charges and a corresponding voltage output. The resulting voltage increases with bending angle, enabling direct measurement of joint motion in applications such as robotic fingers and human limb monitoring.

A distinctive feature of the proposed design is its ability to retain the generated voltage for several seconds after the bending motion ceases. This occurs because the triboelectric charges remain electrostatically separated, causing the TENG structure to exhibit capacitive behavior when connected to a high-impedance load. As a result, the sensor can provide information not only during motion but also under quasi-static conditions, allowing sufficient time for signal acquisition and processing. This preliminary study, therefore, demonstrates the feasibility of a stretchable, wearable, and potentially self-powered TENG-based sensing platform for future applications in joint-angle monitoring, gait analysis, and rehabilitation support. The primary objective is to establish the viability of the sensing concept and validate its fundamental operating principle.

## 2. Fabrication, Materials and Methods

### 2.1. The Structure of the TENG Stretch Sensor

**Figure 1:**
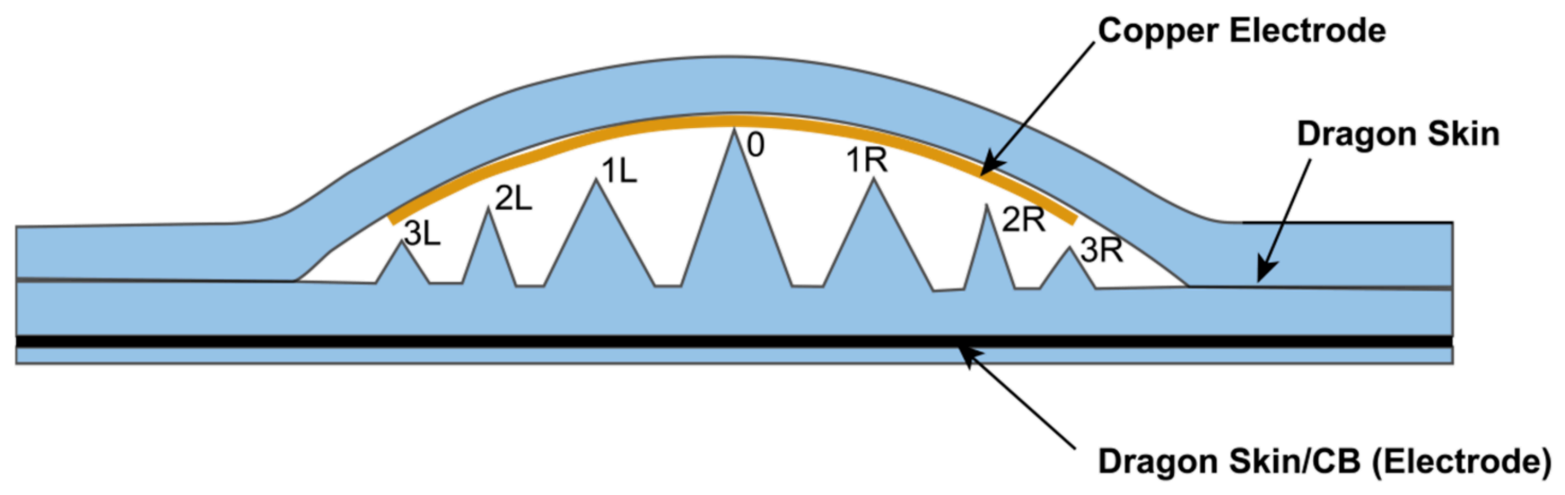
Proposed TENG-based stretch sensor.

The proposed TENG stretch sensor operates through a combination of contact-separation and sliding triboelectric mechanisms and incorporates a unique multi-triangular elastomer structure paired with an arched top electrode, as illustrated in Figure 1. The sensor consists of two main components. The top section comprises a copper layer that serves as both the triboelectric material and the electrical electrode. The bottom section is formed from Dragon Skin silicone, which acts as the negative triboelectric layer.

To enable stretchability, the bottom electrode is fabricated from a conductive carbon black–Dragon Skin composite, allowing it to deform together with the elastomer substrate. For the top electrode, stretchability is achieved through the arched structural design rather than the copper itself. During stretching, the arch gradually flattens, accommodating the applied strain while preventing significant deformation of the copper electrode. This structural approach maintains electrode integrity and electrical performance while allowing the sensor to undergo repeated stretching and bending motions.

### 2.2. The Fabrication of the TENG Stretch Sensor

**Figure 2:**
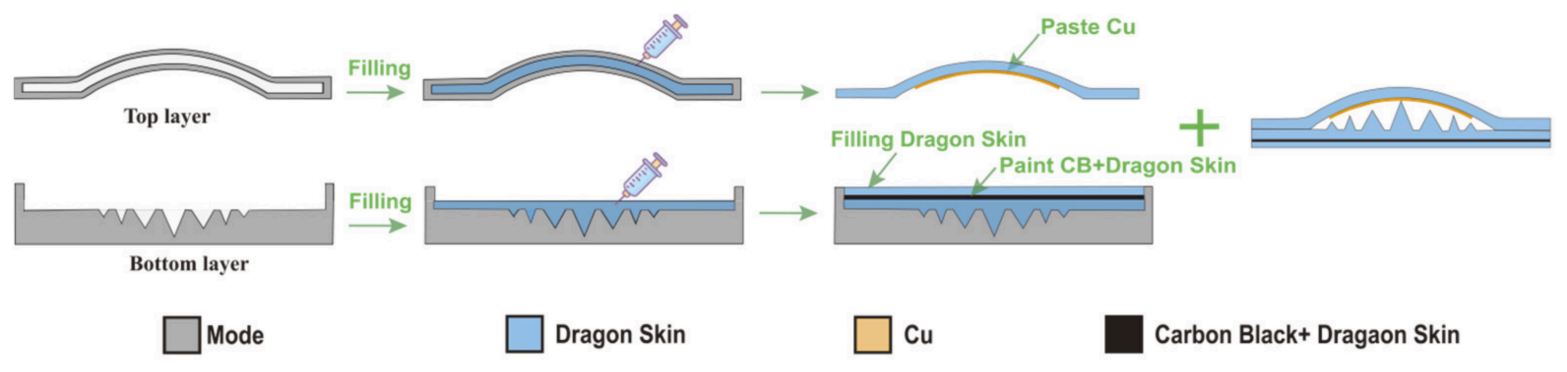
The fabrication process of the TENG-based stretch sensor.

The fabrication process of the proposed TENG stretch sensor is illustrated in Figure 2. The sensor consists of a top arch layer and a bottom stretchable layer. To fabricate the top layer, Dragon Skin silicone (as an elastomer) was poured into an arch-shaped mold and cured to form the encapsulation structure. A copper tape electrode was then attached to the underside of the arch, creating the top triboelectric layer. For the bottom layer, Dragon Skin was cast into a mold containing the multi-triangular features. A stretchable electrode composed of a carbon black–Dragon Skin composite was subsequently incorporated into the structure. After both layers were fabricated, they were assembled using a thin layer of Dragon Skin as an adhesive. The completed sensor has an overall height of approximately 5 mm, with a maximum separation gap of about 3 mm between the two layers. The multi-triangular sensing region measures 12 × 16 mm^2^ and consists of triangular features with heights of 3 mm, 2 mm, 1.5 mm, and 0.75 mm, respectively.

### 2.3. The Working Principle of the TENG Stretch Sensor

As illustrated in Figure 1, stretching the sensor causes the multi-triangular elastomer structure to progressively come into contact with and slide against the copper arch. Contact occurs sequentially, beginning with triangle 0, followed by triangles 1L and 1R, then 2L and 2R, and subsequently triangles 3L and 3R. As deformation increases, both the contact area and sliding interaction between the Dragon Skin and copper surfaces become greater. This enhanced triboelectric interaction generates more surface charges, leading to a higher output voltage. Consequently, the output voltage increases with the degree of stretching, providing a direct means of quantifying sensor deformation. Through this mechanism, the proposed TENG-based sensor can determine the degree of stretching and, therefore, the corresponding joint bending angle from its electrical output.

### 2.4. Experimental Set-Up

**Figure 3:**
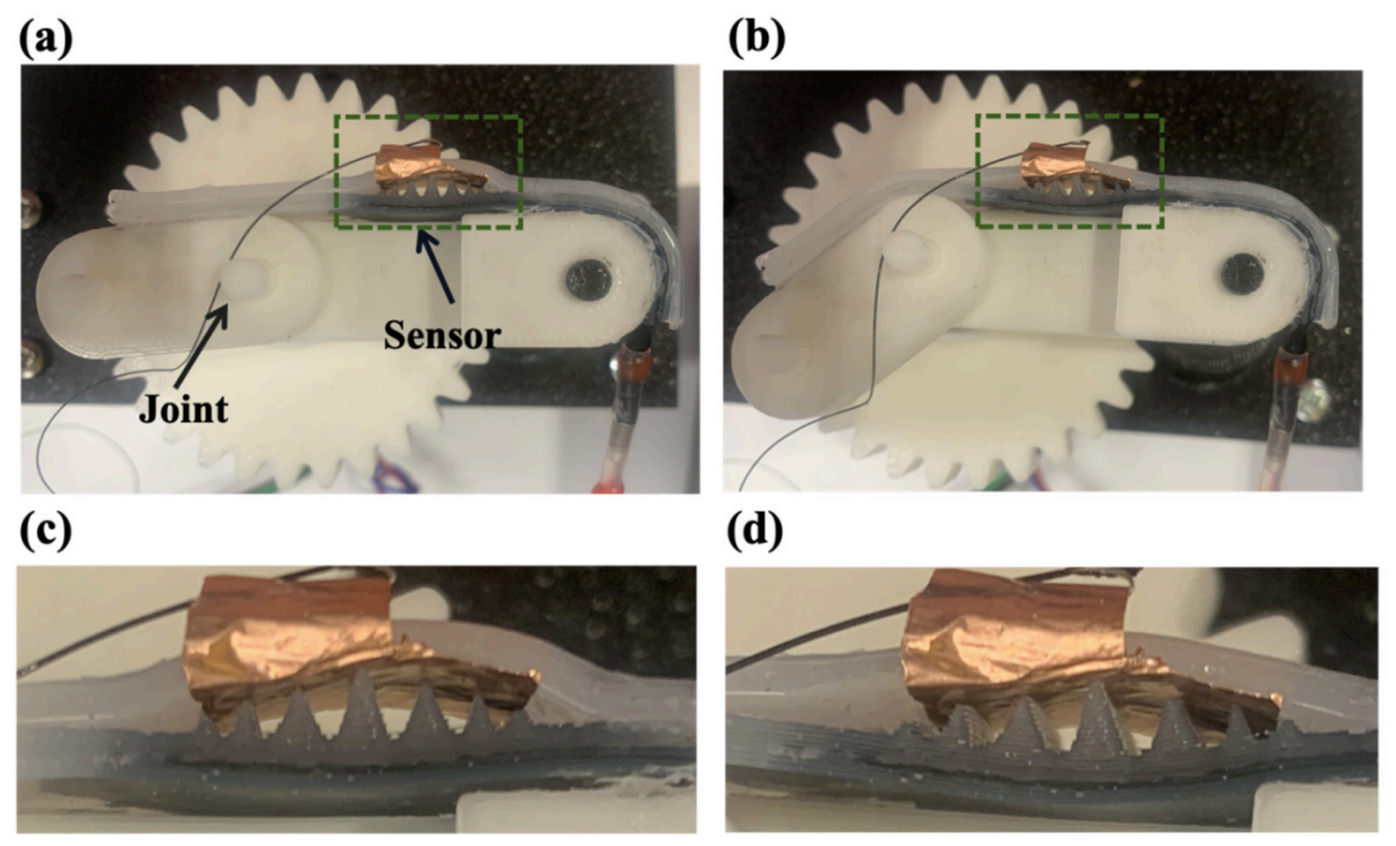
The experimental set-up: (**a**) The sensor is in its original state (unstretched); (**b**) The sensor is rotated to 45° (stretched); (**c**) Enlarged view of sensor in (**a**); (**d**) Enlarged view of sensor in (**b**).

To evaluate the sensor’s suitability for limb-joint and gait-monitoring applications, it was mounted on a simulated joint platform driven by a stepper motor, as shown in Figure 3. This setup enabled precise and repeatable control of the joint bending angle during testing. The sensor’s open-circuit voltage was measured using a Keithley 6517B electrometer (Keithley Instruments, LLC, Solon, OH, USA). All experiments were conducted under controlled laboratory conditions at 22 °C and 55% relative humidity to ensure consistent sensor performance and measurement reliability.

## 3. Results

**Figure 4:**
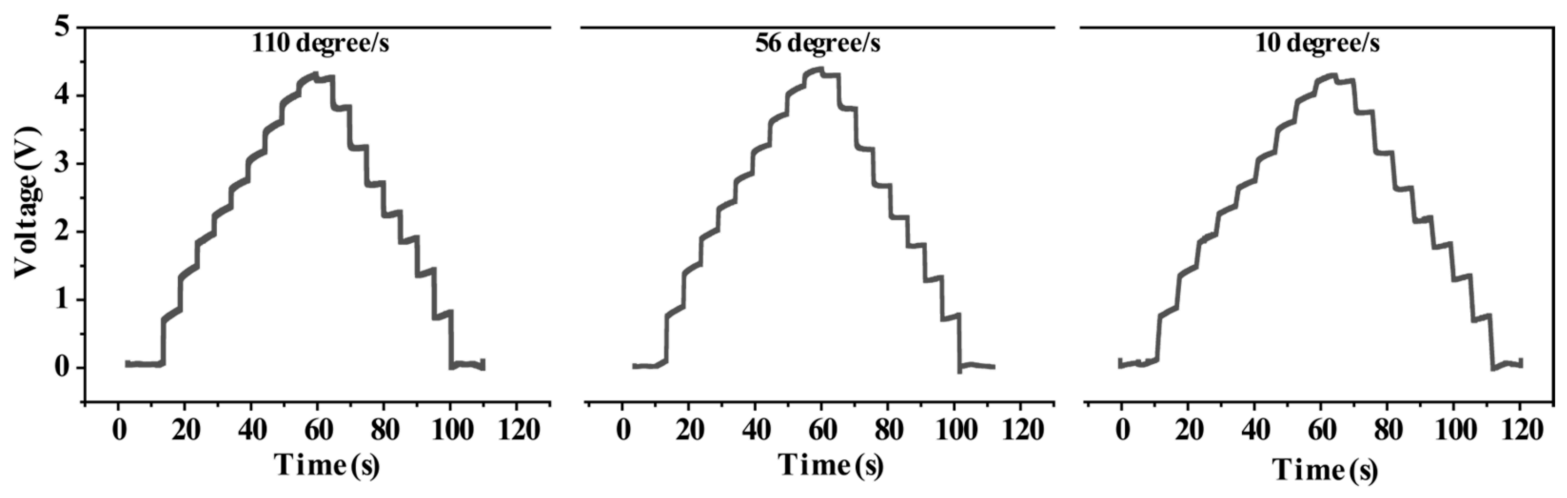
The output of the TENG-based stretch sensors at different stretch rates was recorded by an electrometer with a 10-degree increment.

Figure 4 shows the variation in output voltage as the joint angle increased from 0° to 90°, then returned to its initial position. During the experiment, the motion was paused for 5 s at every 10° increment, and the output voltage was recorded using a Keithley electrometer. Three important observations can be drawn from the results. First, the output voltage increases with bending angle, indicating a clear correlation between sensor output and joint deformation. As the degree of bending increases, the strain also increases, and more sliding occurs between the 2 layers. Hence, the output voltage increases with more sliding. During the reverse cycle, the bending angle decreases, the sliding direction reverses, and the generated voltage has the opposite polarity, thereby reducing the output voltage previously generated during the forward cycle. Second, similar voltage responses were obtained at rotation speeds of 110°/s, 56°/s, and 10°/s, suggesting that the sensor output is largely independent of bending speed within the tested range [23]. Third, the 5 s pauses at each angular position were used to emulate quasi-static conditions. The results show that the generated voltage can be sustained for several seconds after motion ceases, enabling the sensor to monitor not only dynamic motion but also quasi-static joint positions. This voltage-retention capability is particularly beneficial for wearable motion-monitoring applications, where joint angles often need to be measured during both movement and stationary postures.

**Figure 5:**
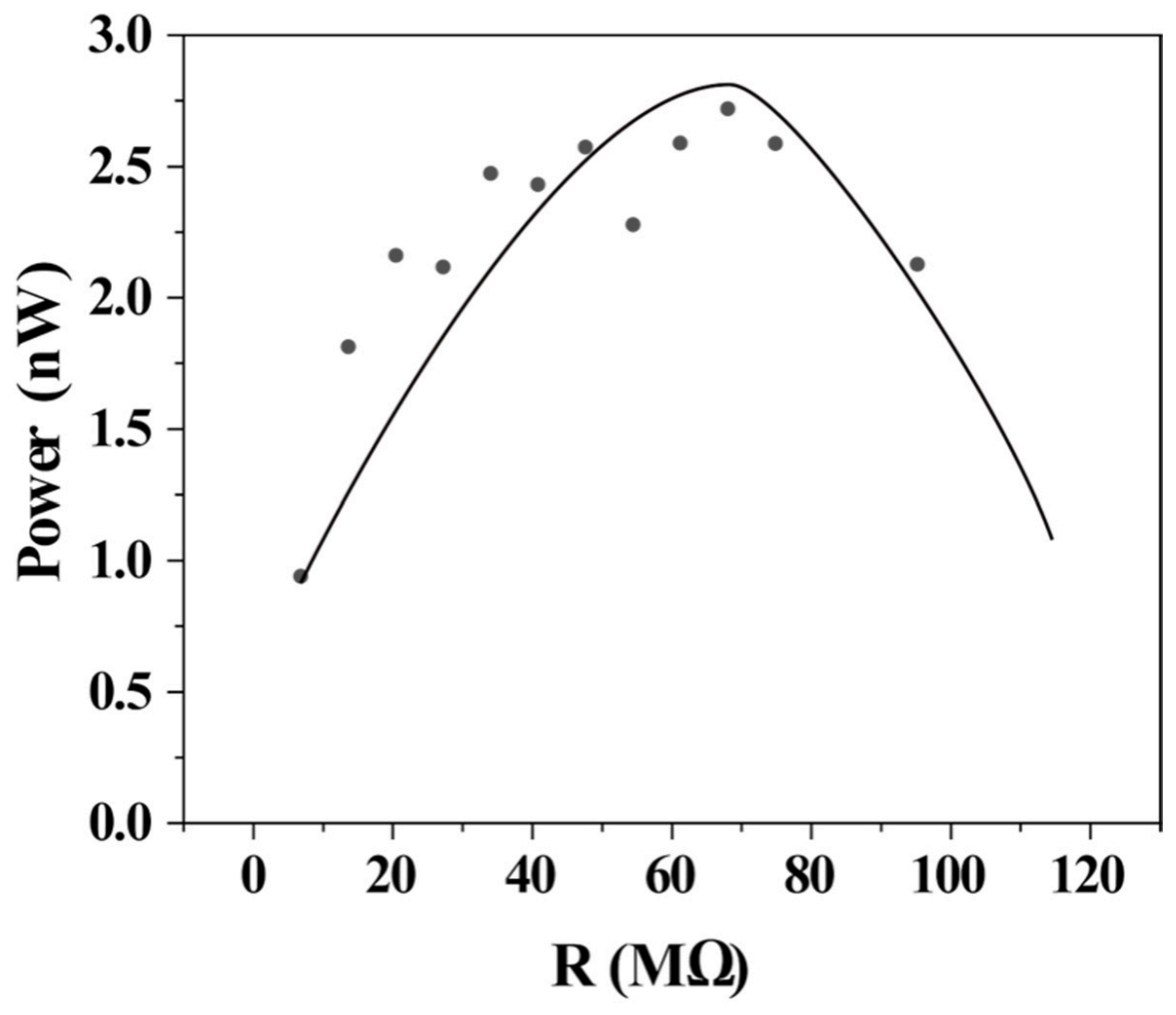
Power versus load resistance across the TENG-based sensor’s output to estimate the TENG’s internal impedance.

Determining the internal impedance of the proposed TENG sensor is important for designing an effective signal-acquisition interface and ensuring proper impedance matching. Figure 5 shows the approximate relationship between output power and load resistance, from which the sensor’s internal impedance was estimated at approximately 66 MΩ. This is merely to demonstrate, not to quantify, the sensor’s impedance, which is high and hence requires proper impedance matching with the electronic circuitry. It should be noted that the measurements presented in Figure 4 were obtained using a Keithley electrometer with an input impedance of approximately 200 TΩ. While this configuration is ideal for sensor characterization, it does not accurately represent practical operating conditions, where the sensor would be connected to a portable electronic data-acquisition system.

**Figure 6:**
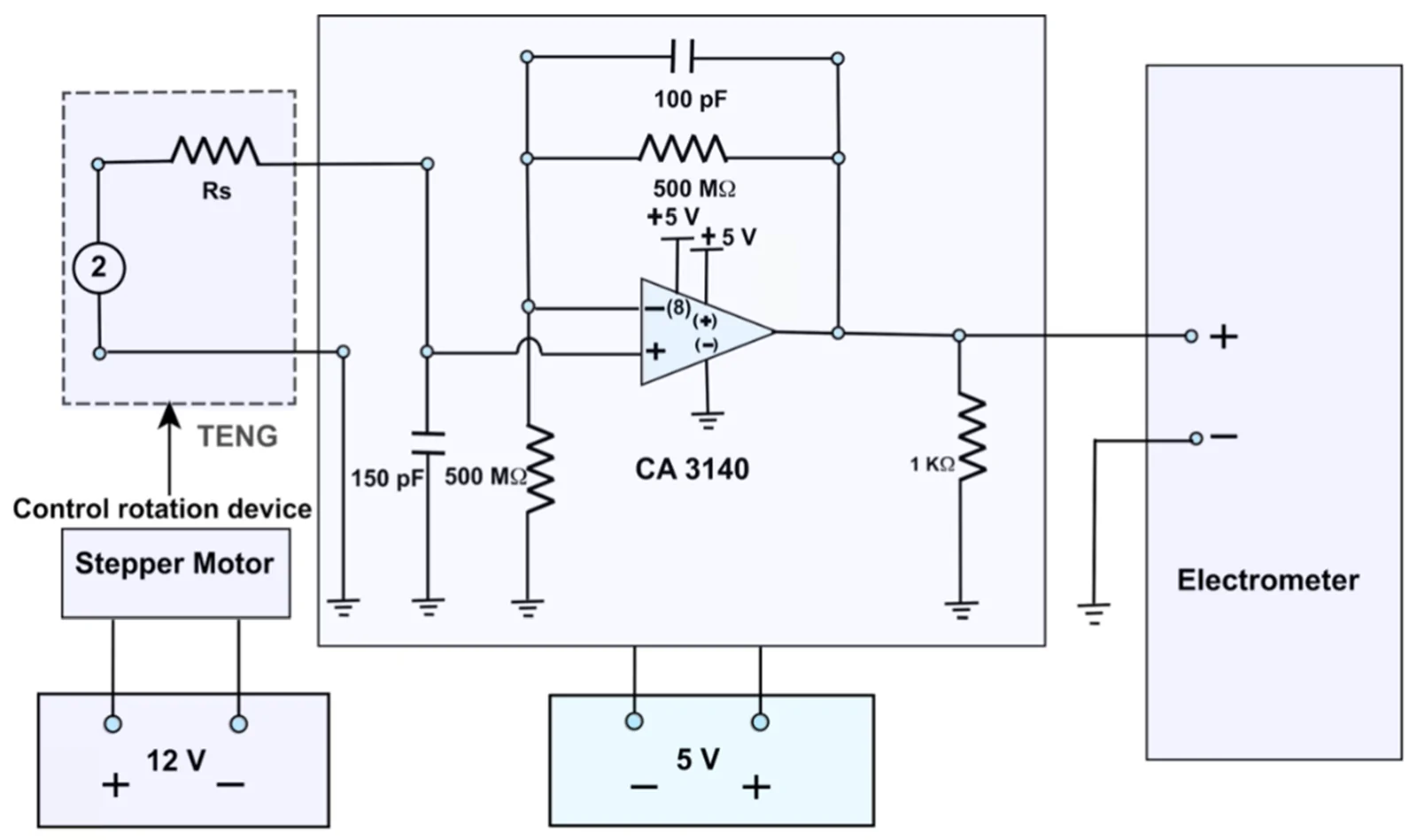
An impedance-matching circuit to interface with the sensor.

For wearable and portable applications, an ESP32-S3 microcontroller (Espressif Systems, Shanghai, China) is proposed as the processing platform. However, the relatively low input impedance of the ESP32-S3 (approximately 1 kΩ) is incompatible with the TENG’s high output impedance. Therefore, an impedance-matching circuit was developed, as illustrated in Figure 6. A CA3140 operational amplifier (Renesas Electronics Corporation, Tokyo, Japan), which provides a very high input impedance of approximately 1.5 TΩ, was used as a buffer stage to bridge the impedance mismatch between the TENG sensor and the microcontroller. For characterization purposes, a 1 kΩ resistor was used to emulate the input impedance of the ESP32-S3. An electrometer was connected across this resistor to measure the buffered output voltage and evaluate the impedance-matching circuit’s performance.

**Figure 7:**
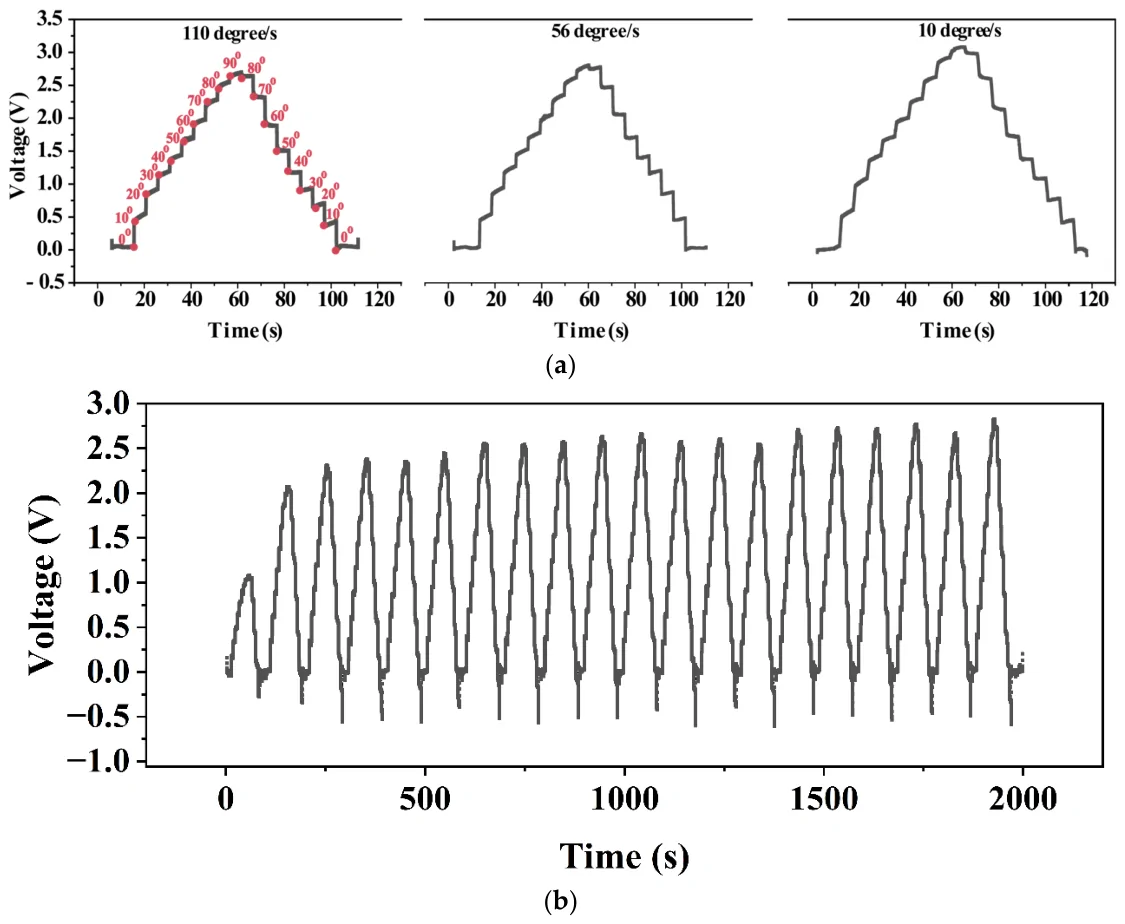
Voltage output during the rotation process. (**a**) 10-degree increments at 5 s intervals (note the corresponding angles in the plot); (**b**) output voltage over 20 cycles from 0° to 90°, across the 1 kΩ.

Figure 7a shows the output voltage measured across the 1 kΩ load resistor connected to the output of the CA3140 impedance-matching circuit at different bending angles and stretching rates. With the impedance-matching circuit in place, the maximum output voltage at a bending angle of 90° decreases from approximately 4 V to 2.75 V. The 5 s holding period was introduced to simulate a static condition, with the estimated maximum holding time being approximately 7.5 s. As shown in Supplementary Information S1, when the sensor was bent to 90°, the output voltage increased abruptly from 0 V to approximately 2.7 V. The voltage then rose gradually to approximately 2.75 V over the next 3 s, likely due to the structure’s mechanical settling. After reaching this peak value, the output voltage remained relatively stable until it began to decay below 2.7 V at approximately 7.5 s, due to charge dissipation within the triboelectric system. The theoretical voltage gain of the impedance-matching circuit is 0.88, indicating that the circuit output voltage is expected to be lower than the true open-circuit voltage measured by the electrometer. A detailed derivation of the theoretical voltage gain of 0.88 for the impedance-matching circuit is presented in Supplementary Information S2. However, the prototype circuit exhibited an actual voltage gain of 0.68. This discrepancy is likely attributable to non-idealities associated with the breadboard implementation, including parasitic capacitance, leakage currents, contact resistance, and other prototyping imperfections. Importantly, this output voltage falls within the 3.3 V input range of the proposed ESP32-S3 microcontroller, enabling direct signal acquisition without additional voltage-conditioning circuitry. Furthermore, the voltage profiles obtained using the impedance-matching circuit closely resemble those measured with the high-impedance electrometer, confirming that the circuit effectively preserves the sensor response while providing a practical interface for portable electronics.

Figure 7b presents the cyclic performance of the sensor during repeated bending and release, in which the joint angle was varied from 0° to 90° in 10° increments, with a 5 s pause at each position. The test was conducted over 20 consecutive cycles. The results show that the output voltage gradually increases during the initial cycles, then stabilizes after approximately 10 cycles. This behaviour is a known characteristic of TENG devices and can be attributed to a preconditioning effect, whereby triboelectric charges accumulate progressively during repeated operation until a steady-state charge density is established [24]. Note that the results presented in Figure 4 and Figure 7a were obtained after the preconditioning. Once this equilibrium is reached, the sensor exhibits a stable and repeatable output, demonstrating its suitability for continuous joint-motion monitoring applications.

**Figure 8:**
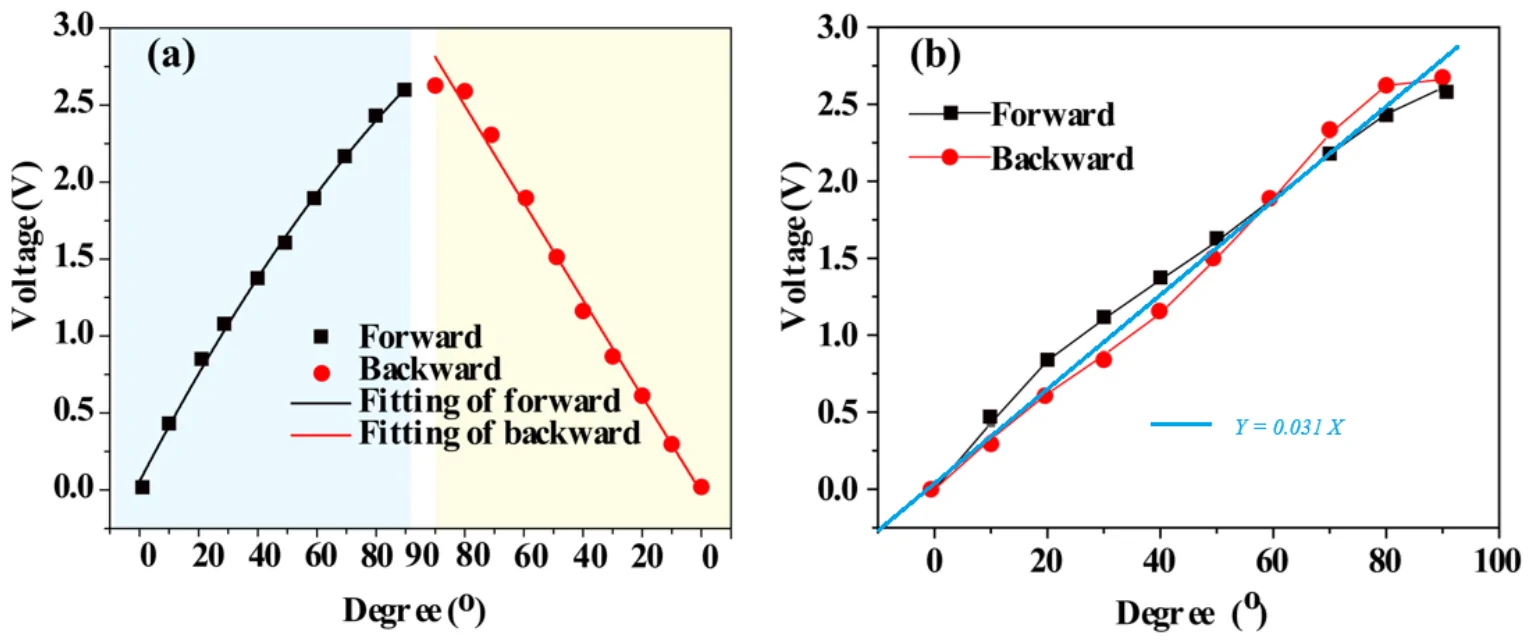
Output voltage versus bending angle. The output voltage is measured at (**a**) 10-degree intervals (**b**), the estimated hysteresis of 8.6%.

Figure 8a presents the output voltage measured across the 1 kΩ load resistor at 10° bending increments during both forward and reverse rotations at a speed of 110°/s. These results are replotted in Figure 8b to highlight the relationship between the output voltage and bending angle. A clear, nearly linear correlation is observed, demonstrating the sensor’s capability for accurate angle measurement. Based on the slope of the voltage-angle curve, the sensor exhibits a sensitivity of approximately 31 mV/°, while maintaining a relatively low hysteresis of about 8.6% between the loading and unloading cycles. When the joint is bent to 90°, the corresponding strain experienced by the sensor is approximately 0.45. These results indicate that the proposed TENG-based stretch sensor can track joint motion with high sensitivity and repeatability over a practical range of deformation.

## 4. Discussion and Conclusions

This preliminary study presents a TENG-based stretch sensor that combines a multi-triangular elastomer structure with an arched copper electrode, and its viability as a stretch sensor has been demonstrated. When the elastomer is stretched during joint bending, the triangular features slide against the copper surface, generating triboelectric charges and producing a measurable voltage output. Testing on a simulated joint platform showed a clear relationship between the output voltage and the bending angle, demonstrating the sensor’s ability to monitor joint movement effectively.

As the bending angle increases, the contact and rubbing between the elastomer and copper surfaces become more pronounced, generating a larger triboelectric output. This leads to a corresponding increase in voltage, allowing the bending angle to be estimated directly from the sensor response. These results confirm that the proposed TENG-based sensor can function as a practical stretch sensor for joint-angle measurement. However, it is important to note that the proposed sensor does not measure the absolute angle. Instead, it provides a relative measurement, as each increment in output voltage is dependent on the preceding output value. To facilitate integration with portable electronics, an impedance-matching circuit is proposed to ensure that the maximum output voltage at a bending angle of 90° remains within the proposed ESP32-S3 microcontroller’s 3.3 V input range. This enables direct signal acquisition without complex signal-conditioning circuitry, supporting the development of compact and wearable sensing systems.

A particularly interesting feature of the proposed sensor is its ability to maintain the generated voltage for several seconds after the bending motion has stopped. This occurs because the triboelectric charges remain electrostatically separated, causing the TENG structure to behave like a capacitor when connected to a high-impedance load. As a result, the stored charge dissipates slowly, allowing the voltage to be retained long enough for the microcontroller to capture and process the signal. This characteristic enables the sensor to detect not only dynamic movements but also quasi-static joint positions.

**Figure 9:**
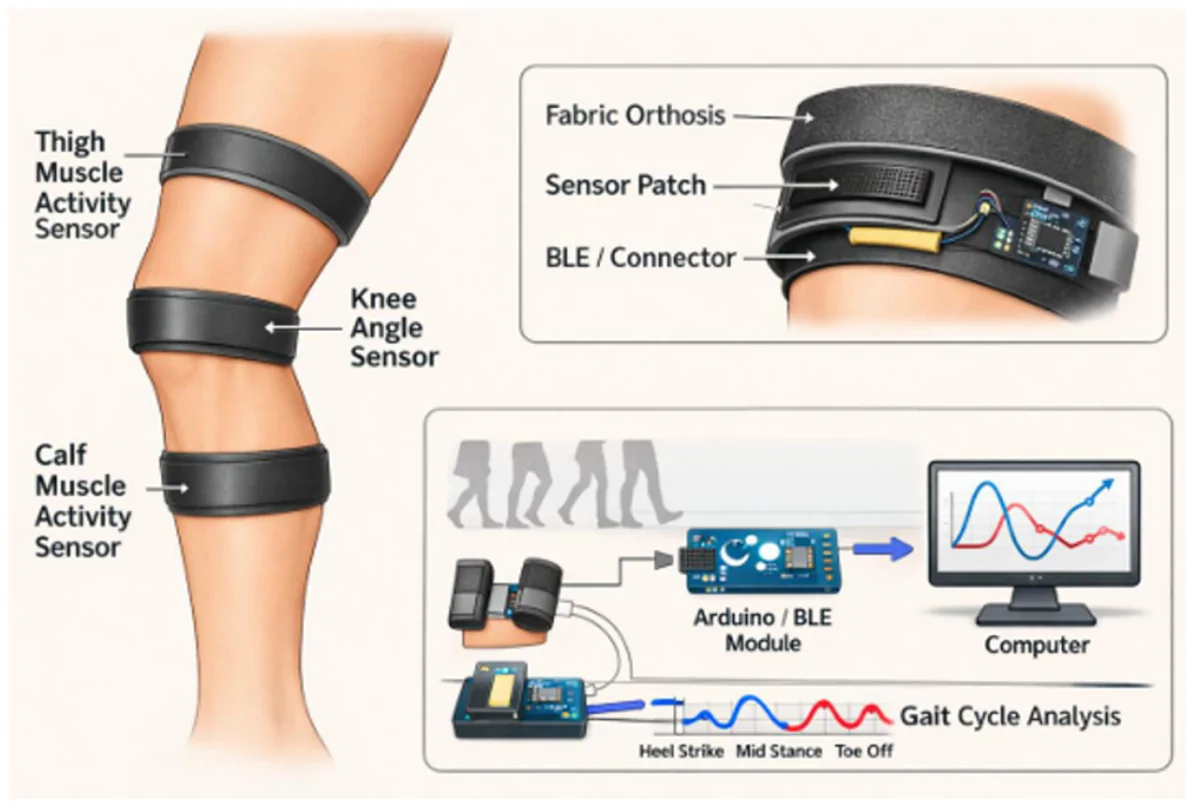
A proposal for the application of the TENG-based sensor for monitoring walking gait (AI-generated).

Overall, the findings demonstrate that TENG technology can be successfully applied to stretch sensing and joint-angle monitoring. The prototype sensor achieved a sensitivity of approximately 31 mV/°, which, when combined with the 12-bit analog-to-digital converter of the ESP32-S3, is expected to provide sufficient resolution for practical motion-monitoring applications. This work serves as an important first step toward developing stretchable, wearable, and potentially self-powered sensing systems for human motion analysis. In future work, the sensor will be further optimized and integrated with ESP32-S3 into wearable platforms for lower-limb gait monitoring and rehabilitation applications, as illustrated in Figure 9.

Although the proposed sensor demonstrates encouraging performance, further work is required to fully assess its practical applicability. Future studies will investigate its long-term stability, durability, and reliability under prolonged operation. Attention will be given to the effects of temperature and humidity variations, material fatigue resulting from repeated deformation, and performance degradation over time. These investigations will provide a more comprehensive understanding of the sensor’s suitability for wearable motion-monitoring and rehabilitation applications.

## Data Availability

The raw data supporting the conclusions of this article will be made available by the authors on request.

## Supplementary Materials

The following supporting information can be downloaded at https://www.mdpi.com/article/10.3390/s26206493/s1. The following supporting information is available: Raw data in an Excel file and Supplementary Information.
