# Supplementary material for: Triboelectric Nanogenerator-Based Stretch Sensor
Source: Sensors (Basel). 2026 Oct 14;26(20):6493. doi: 10.3390/s26206493 (PMC13709237; doi:10.3390/s26206493)
Supplement: Supplementary file 1 [file sensors-26-06493-s001.zip › sensors-4609238-supplementary.pdf]

S1

The plot below shows the sensor output voltage over time as the sensor was rotated from 0° to 90°. As the sensor was stretched during the rotation, the output voltage increased from 0 V to approximately 2.7 V. However, after reaching 90° and being held in that position, the voltage continued to increase slightly, reaching approximately 2.75 V after 3 s, then gradually decreasing to approximately 2.7 V at around 7.5 s. It is hypothesised that the triangular structure did not become completely mechanically stable immediately upon reaching 90°, resulting in a small continued increase in voltage during the first few seconds. After this period, charge dissipation within the triboelectric system likely became dominant, leading to the observed gradual decrease in voltage.

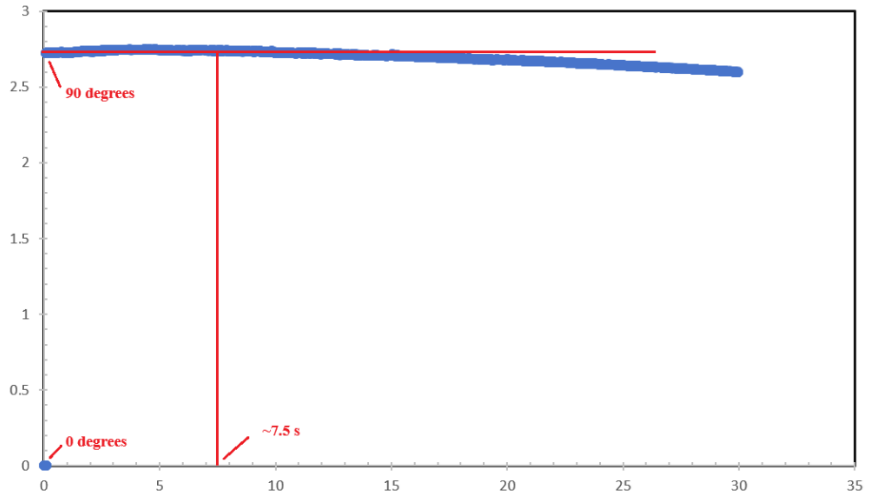

S2

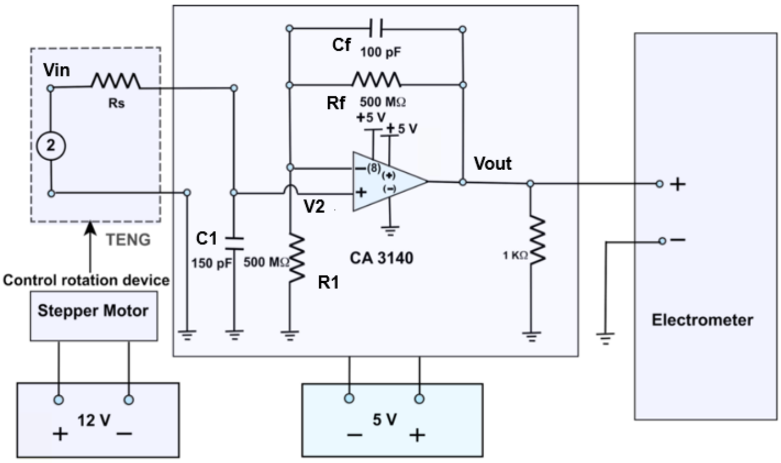

$$\frac{V_{in} - V_2}{R_s} = \frac{V_2}{R_1} + V_2.(sC_1)$$

$$\frac{V_2}{V_{in}} = \frac{1/R_{in}}{1/R_s + 1/R_1 + s.C_1}$$

Substituting the components value.

$$= \frac{1/66 \times 10^6}{1/66 \times 10^6 + 1/500 \times 10^6 + s(150 \times 10^{-12})}$$

As it is operating in DC mode, s = 0 and

$$\frac{V_2}{V_{in}} = \frac{1.515}{1.715} \approx 0.88$$

$$\frac{V_{out} - V_2}{Z_f} = I_{in-}$$

Assuming $I_{in-} = 0$, since the op-amp has extremely high input impedance.

$$\frac{V_{out} - V_2}{Z_f} = 0$$

Supplementary Information

$$V_{out} = V_2$$

$$\frac{V_{out}}{V_2} = 1$$

$$\therefore \boldsymbol{\frac{V_{out}}{V_{in}} = \frac{V_{out}}{V_2} \times \frac{V_2}{V_{in}} = 0.88}$$
